# Open Access Data Sharing in Genomic Research

**DOI:** 10.3390/genes5030739

**Published:** 2014-08-29

**Authors:** Stacey Pereira, Richard A. Gibbs, Amy L. McGuire

**Affiliations:** 1 Center for Medical Ethics and Health Policy, Baylor College of Medicine, Houston, TX 77030, USA; E-Mail: spereira@bcm.edu; 2 Human Genome Sequencing Center, Baylor College of Medicine, Houston, TX 77030, USA; E-Mail: agibbs@bcm.edu

**Keywords:** data sharing, genomics, privacy, genetic discrimination, open access database

## Abstract

The current emphasis on broad sharing of human genomic data generated in research in order to maximize utility and public benefit is a significant legacy of the Human Genome Project. Concerns about privacy and discrimination have led to policy responses that restrict access to genomic data as the means for protecting research participants. Our research and experience show, however, that a considerable number of research participants agree to open access sharing of their genomic data when given the choice. General policies that limit access to all genomic data fail to respect the autonomy of these participants and, at the same time, unnecessarily limit the utility of the data. We advocate instead a more balanced approach that allows for individual choice and encourages informed decision making, while protecting against the misuse of genomic data through enhanced legislation.

## 1. Introduction

Last year marked the 10th anniversary of the completion of the Human Genome Project (HGP) [1]. One of the many accomplishments of the HGP was the broad sharing of data generated by genomic research studies in order to maximize the utility of the data and the public benefit of such projects [2]. This helped to create a culture of openness in genomic research that was codified in a joint policy from the National Human Genome Research Institute (NHGRI) and the Department of Energy (DOE) in 1991 [3] that called for the rapid public release of data generated by the HGP and subsequent projects. Additional policies in the following years, both domestic and international, reaffirmed and expanded these recommendations for publicly sharing large-scale DNA sequence data [4,5,6,7].

Initially, the means for protecting participants’ privacy when these data were shared in open access (publicly accessible) databases rested upon the “de-identification” of the data by stripping them of all recognizable annotation before sharing. DNA has a very high information content, however, and in 2004, Lin *et al.* showed that it is possible to identify single individuals with as few as 30–80 single nucleotide polymorphisms (SNPs) [8,9], prompting new privacy concerns. In 2006, the U.S. National Institutes of Health (NIH) established the Database of Genotypes and Phenotypes (dbGaP) [10], which is a controlled access database, meaning that individual level genetic data are accessible only with approval from a Data Access Committee. The current NIH data sharing policy requires researchers to obtain approval from their institution before sharing genomic data in dbGaP, and provides guidance to institutions on how to review studies to ensure compliance with the policy, particularly with regard to the adequacy of informed consent documents.

In 2008, Homer *et al.* revealed further complications by showing that it was possible to uniquely identify individuals in aggregated data sets [11]. This led to the implementation of additional protections by restricting access to some aggregated data elements in dbGaP and other databases internationally [12]. Further, in early 2013, Gymrek *et al.* demonstrated that it was possible to identify individuals in the open access database of the 1000 Genomes Project by analysis of Y-chromosome short tandem repeats. They compared these data to genetic information available on a recreational genealogy website, and then used that information to link to additional publicly accessible data, such as obituaries and the National Institute of General Medical Sciences (NIGMS) Human Genetic Cell Repository, which banks samples from one of the same populations that took part in the 1000 Genomes Project [13]. This paper was the first to show unequivocally that individuals could be uniquely identified without first obtaining a reference sample. In response, the NIH worked with the NIGMS to move age information, which was previously publicly accessible, into the controlled-access part of the database [14].

Each successive policy decision to further restrict access to genomic data has received some pushback, with critics arguing that each was an overreaction and would unnecessarily impede science [12]. Nonetheless, limiting access to increasing amounts of data continues to be the primary policy response to mounting privacy concerns. Arguments against restricted access and for more open data sharing policies must balance the social and scientific benefits of unrestricted access to and use of data, with adequate protection of the rights and interests of individuals who contribute biological specimens and information to research. The almost exclusive focus on restricting access to genomic data as a matter of policy, however, impedes research and fails to respect the autonomy of those who choose to share their information openly. It has been observed that data in controlled access databases are used less frequently than data in open access databases, and as Rodriguez *et al.* [14] remind us, researchers and other custodians have an ethical responsibility not only to minimize the risk of harm to participants, but also to maximize the utility of generated data. These considerations have led some groups to advocate for a more balanced approach that expands options for open access genomic data release [15,16]. Providing research participants the opportunity to allow their data to be shared more broadly is consistent with the principle of respect for autonomy [9], and as we show below, at least among certain populations, there are a considerable number of “information altruists” [17] who would agree, if given the choice.

## 2. Participant Perspective

Although studies suggest that there is significant public concern about genetic privacy [18,19], in at least one study, the majority (60%) of more than 4600 U.S. adults surveyed reported willingness to participate in genomic research [20]. Likewise, we have found that a substantial number of research participants are even willing to consent to open access release of their genomic data. In a randomized trial of consent with 323 genomic research participants, the majority (84%) agreed to open access data release. Even after being debriefed, educated about all of the consent options, including the option to consent only to the release of data into controlled access databases like dbGaP, or not at all, and surveyed about their perspectives and concerns, the majority (53%) chose to allow their data to be shared in open access databases [21].

We found a similar response from participants in the Texas Cancer Research Biobank (TCRB), which aimed to establish a fully functional open access database incorporating cancer genomes and other participant data. Controlled access data release was a condition of participation in the TCRB, but the informed consent process allowed participants to opt in to broader sharing of their genomic information via open access data release. Of the 194 participants who were offered this choice, 122 (63%) agreed to open access data release.

These studies present an encouraging picture of research participants’ altruistic motivations and lend support to the argument that restrictive data sharing policies fail to respect autonomy of participants who would choose to make their data more broadly available. However, they also raise two major challenges that deserve careful consideration: (1) genomic data sharing is a complex concept that can be difficult for participants to understand; and (2) there is a diversity of perspectives about open access data sharing and certain groups may be less willing to share their data publicly.

### 2.1. Participant Understanding

Autonomous decision-making requires adequate understanding of the options presented. Yet, ensuring adequate understanding is a challenge in all research involving human subjects. Studies suggest that research participants generally have difficulty understanding and remembering basic information described in research-informed consent documents (e.g., the purpose and risks of the research, as well as general concepts related to study design, like randomization) [22,23]. Genomic research and data sharing are complex concepts, so it is not surprising that participants also have difficulty understanding the differences between data sharing options. For example, in the randomized trial of consent mentioned above, a majority (54%) of participants who were surveyed either could not initially recall with whom they had agreed to share their data or did not understand that by agreeing to open access data sharing it meant that their data could be accessed and used by anyone on the internet without restriction [24]. One possible solution is to try to improve understanding with targeted educational interventions, such as brochures or videos. However, studies have shown that efforts to improve understanding have had only limited success, with the most effective intervention being on-on-one education [25,26].

Another approach to ensure participant awareness is to only release data into open access databases when participants can directly exhibit adequate understanding. For example, the Personal Genome Project, which aims to create a publicly available database of genomic and health information with no expectations of privacy, requires participants to correctly answer all questions in an enrollment examination prior to being allowed to participate, although they may retake the examination multiple times [27]. Similarly, in the TCRB, mentioned above, a subset (*n* = 37) of participants who had agreed to open access data sharing took part in an education session that described the difference between controlled and open access data release and the risks and benefits of each in a question and answer format with visual aids as appropriate. After completing the education session, participants were asked to take a survey, one aim of which was to assess understanding. We found that 73% of survey participants demonstrated adequate understanding, which we defined as (1) knowing that they agreed to open access data sharing; (2) knowing who could access data in an open access database; and (3) understanding the risk of discrimination associated with open access data sharing. Only data from those who had demonstrated adequate understanding were eligible for open access data release. We also assessed participants’ risk tolerance and decisional conflict. Fifty-four percent of participants reported high risk tolerance, meaning that they (1) were comfortable sharing their genetic and health information with the general public; and (2) would still participate even if they knew someone would identify their genetic data. Using an adapted version of the decisional conflict scale [28], we found that 68% demonstrated low decisional conflict, meaning that they answered all six questions in a manner indicating that they had low decision uncertainty, no pressure from others, and high perceived effective decision making. Fourteen participants (38%) changed their consent and refused open access data sharing at the completion of the education session. Of the 23 participants (62%) who still agreed to open access data release, 19 had adequate understanding and were therefore eligible for participation. Data from those with high risk tolerance and low decisional conflict were prioritized for public release.

There is considerable debate elsewhere concerning the definition of adequate understanding in research and how best to measure it [29]. Some have focused on developing educational interventions, such as those mentioned above, while others have proposed simplified consent documents as a way to improve understanding [30]. In the PGP and the TCRB, extensive measures were taken to assess understanding and to release data only from those who met a predefined threshold of comprehension. This is time consuming and resource intensive and may not be feasible in all genomic studies. Additional research is needed to identify methods of measuring and improving understanding that are not only effective, but are also efficient, especially in the context of genomic research involving open access data sharing. This is particularly important because participants’ right to withdraw from the research is necessarily limited by the inability to retrieve data that has been shared publicly. As these studies suggest, however, there is a subset of participants who understand the implications of open access data release and voluntarily agree to it.

### 2.2. Diverse Viewpoints

It is important to note that the participants in both the randomized trial of consent and the TCRB were primarily quite ill (sometimes with terminal disease), very trusting of their physicians, and highly motivated to participate in research. Even among this group, however, there was diversity of perspectives about open access data sharing. In the randomized trial of consent, for example, Hispanic, unmarried, and more educated participants were all less likely to choose public data release, as were parents who were making decisions about the release of their child’s data [21].

Other populations may exhibit even more variation in their perspectives on data sharing. For example, Lemke *et al.* [31] explored public and biobank participants’ attitudes toward genomic research and data sharing via focus groups. While different levels of data sharing (*i.e.*, open *versus* controlled access) were not specifically examined in those studies, the investigators found more generally that there was wide variation in views on genomic data sharing, with some study participants more comfortable than others. Similarly, Trinidad *et al.* [32] conducted focus groups with research participants, surrogate decision-makers, and members of a health maintenance organization to investigate perspectives toward data sharing. They also found that perspectives varied, although they report that study participants were generally supportive of genomic data sharing for scientific benefit. In a commentary on conducting research with tribal communities in the U.S., Harding *et al.* [33] argue that special considerations that take into account the populations’ perspectives are important when developing data sharing agreements with Native American tribes.

Our focus in this paper is data sharing in the context of the United States. Research participants in other parts of the world may feel differently about their genomic data and whether or not it should be shared for research purposes [34]. Thus, although generally reported as positive, participant perspectives on data sharing vary between populations, as well as among individuals, based on context, clinical circumstances, and personal values and beliefs.

## 3. Toward a More Balanced Approach

The variation in individual and group preferences for and understanding about genomic data sharing suggests that both mandatory public data release, as well as blanket restriction of access to genomic research data as a matter of policy, are misguided. Regulatory bodies in general tend to address this “heterogeneity problem” by taking the most restrictive and risk averse approach [35], which, in this case, inhibits choice by prohibiting the broader release of data from those who understand and are comfortable with open access sharing. It also reportedly impedes research [14], although studies quantifying the added benefit of open access *versus* controlled access data sharing are required. We advocate instead for a more balanced approach that allows for individual choice, but provides protection to participants by supporting adequate understanding as part of the informed consent process, and by strengthening accountability and protections against the misuse of available data.

Recent accounts demonstrate that some sophisticated patients are exercising their autonomy by sharing data themselves using existing platforms, such as social media, in order to facilitate discovery for rare and serious diseases [36]. If people are to share their own data, it is important that they are aware of the risk of identifiability and understand the challenge of obscuring segments of data in the context of public release [37]. For those whose data are shared within the research context, novel approaches have been suggested to give participants more control over decisions about who can access their data, as well as the ability to continue to manage such choices. For example, a relatively new platform called Reg4All [38] facilitates the sharing of health information in order to find relevant clinical trials, but also gives its users the ability to make finely-tuned choices about who can access their information or contact them. Others have introduced new approaches to consent that allow participants to be more nuanced in their choices, as well as change those choices over time [39], though, arguably, once data are released in an open access manner, there is no way to guarantee their removal from the public domain.

Increased participant engagement and open access data sharing could both be accomplished with modifications to the existing dbGaP model. As it is currently designed, all individual level genomic data in dbGaP is accessible only via controlled access [10]. The NIH could support more broad sharing by creating a publicly accessible segment of dbGaP that includes data from those who agree to open access data release. Participants could also be provided the option for open access data sharing in the informed consent document when agreeing to participate in NIH-funded genomic research. If a participant changes her consent over time, a request could be made to dbGaP to move the relevant data from the open access portion of the database to controlled access.

Regardless of mechanism, if genomic data are made publicly available, then the individuals from whom those data originate ought to be protected against the misuse of that information. One way of providing some protection for these participants could be the use of “click-through” data use agreements. In this model, the person accessing the data would have to read and agree to a list of conditions of use of the data, including agreeing to not attempt to identify the individuals from whom the data came. However, while this may require those accessing the data to recognize that attempting identification would be a violation of the use of the data, such click-through data use agreements are not enforceable, and as such, may not provide adequate protection.

There are existing laws in the United States that provide protection against misuse of genetic information. The vast majority of states have laws that govern the use of genetic information in health insurance and employment [40]. Likewise, the Genetic Information Nondiscrimination Act (GINA) [41], in effect as of 2009, makes it illegal for health insurers and employers with 15 or more employees to discriminate against people based on their genetic information. GINA has both corrective and monetary penalties that vary based on the intention and severity of the infraction. However, it does not protect against genetic discrimination in other types of insurance, such as long-term, disability, and life insurance, or any other realm outside of health insurance and employment. Additionally, some report not feeling fully protected by GINA, leading some to decline acceptance of DNA sequencing in both clinical and research-related contexts for fear of discrimination [42]. In contrast, the Human Tissue Act of the Parliament of the United Kingdom [43], which regulates activities with human bodies and tissues and also provides protection against the use of DNA without consent, is not limited to such contexts, and carries criminal penalties for violations that range from a fine to up to three years in prison. Though criminal law may not be the best approach to discourage the misuse of genetic data in the U.S., stricter penalties and broader protections against misuse of data by any third party may be needed to protect individuals who agree to share their data broadly for the public’s benefit.

## 4. Conclusions

In the context of research, investigators have a professional obligation to be good stewards of the data with which research participants have entrusted them. In order to fulfill this obligation, we need policies that respect participant autonomy and maximize the utility of the data, alongside strengthened legislation that protects those participants from the misuse of their genomic information. The field has made great progress in the 10 years since the completion of the Human Genome Project. We must find ways to protect participants, yet avoid unneeded hindrances of researchers’ access to genomic information.
